# Epidemiological modelling of the health and economic effects of COVID-19 control in Australia’s second wave

**DOI:** 10.1007/s10389-021-01611-0

**Published:** 2021-06-28

**Authors:** R. Quentin Grafton, John Parslow, Tom Kompas, Kathryn Glass, Emily Banks

**Affiliations:** 1 Crawford School of Public Policy, The Australian National University, Canberra, Australian Capital Territory Australia; 2 Hobart, Australia; 3 Centre of Excellence for Biosecurity Risk Analysis, School of Biosciences, University of Melbourne, Melbourne, Victoria 3010 Australia; 4 College of Health and Medicine, The Australian National University, Canberra, Australian Capital Territory Australia

**Keywords:** Pandemic, SARS-CoV-2, Individual based models, Compartment models, Social distancing

## Abstract

**Background:**

We investigated the public health and economy outcomes of different levels of social distancing to control a ‘second wave’ outbreak in Australia and identify implications for public health management of COVID-19.

**Methods:**

Individual-based and compartment models were used to simulate the effects of different social distancing and detection strategies on Australian COVID-19 infections and the economy from March to July 2020. These models were used to evaluate the effects of different social distancing levels and the early relaxation of suppression measures, in terms of public health and economy outcomes.

**Results:**

The models, fitted to observations up to July 2020, yielded projections consistent with subsequent cases and showed that better public health outcomes and lower economy costs occur when social distancing measures are more stringent, implemented earlier and implemented for a sufficiently long duration. Early relaxation of suppression results in worse public health outcomes and higher economy costs.

**Conclusions:**

Better public health outcomes (reduced COVID-19 fatalities) are *positively* associated with lower economy costs and higher levels of social distancing; achieving zero community transmission *lowers* both public health and economy costs compared to allowing community transmission to continue; and early relaxation of social distancing *increases* both public health and economy costs.

## Introduction

Australia recorded its first case of COVID-19 on 25 January 2020 from a person who had flown from China on 19 January (Hunt 2020). Initial national daily cases peaked at 458 on 28 March 2020 and, thereafter, declined due to border measures for overseas arrivals, self-quarantine, wide-spread testing and contact tracing, and social distancing outside of households that changed the frequency, the numbers and the nature of physical contacts (Australian Department of Health 2020a). By 9 June 2020, there were only two new recorded cases in Australia.

At the end of May, in the Australian state of Victoria, a ‘second wave’ of COVID-19 began following an outbreak from hotel quarantine. Additional public health measures were reinstituted in the second half of June in Victoria, including a ‘stage 3’ lockdown in the state capital, Melbourne, and the nearby Mitchell Shire, on 9 July for a 6-week period. These measures slowed the growth in infections (Saul et al. 2020) but failed to stop an increase in COVID-19 infections. On 5 August 2020, when new daily cases peaked at 687 in Victoria, there were approximately 20,000 reported cumulative cases in Australia, of which some 11,000 had recovered; with 250 COVID-19 fatalities (162 in Victoria) (Australian Department of Health 2020b).

A highly stringent lockdown, with mandated social distancing measures, began in Victoria in early August 2020. These measures were gradually relaxed from mid-September as infections were effectively suppressed (Blakely et al. 2020) followed a ‘Roadmap for Reopening’ pre-determined by the 14-day average of new daily cases (Victorian Department of Health and Social Services 2020). Major relaxation of social distancing measures occurred on 27 October (step 3) and 22 November (last step) (Grafton et al. 2020). On 6 December 2020, Victoria returned to ‘COVID-normal’ because no new cases had been diagnosed since 31 October.

Key public health and economy questions facing Australia, and the world, in relation to supressing COVID-19 infections are: What levels of social distancing are required to adequately reduce infections associated with a ‘second wave’ of COVID-19 infections? What is the probability of achieving elimination (defined as no community transmission (Group of 8 Universities 2020)) with various levels of lockdown and duration? What are the public health and economy costs of different stringency levels of social distancing?

Our contribution is to show: (1) Epidemiological modelling, undertaken at the peak of the second wave, can provide a reasonable approximation of the actual suppression outcomes (cases) associated with highly stringent social distancing; (2) A combination of epidemiological models, coupled with economy cost measures, show that public health outcomes are associated with lower economy costs; (3) Different epidemiological (individual based, deterministic compartment and stochastic compartment) models can be complementary and provide comparable simulated results; (4) the importance of a sufficiently long enough duration of social distancing, if elimination of community transmission is the goal; and (5) the public health dangers of outbreaks from quarantine even with very low levels of occurrence.

## Materials and methods

### Model description

The simulation model used here is one of a suite of epidemiological models developed to support the study and management of the COVID-19 outbreak in Australia. The suite builds on a stochastic individual-based or agent-based model (IBM), which follows infected individuals through multiple stages and alternative fates as the disease progresses. Individual-based models are flexible but are computationally expensive to run when the number of individuals becomes large. This makes IBM more difficult to undertake rigorous statistical calibration, or to run large ensembles to quantify stochastic variation. Thus, we developed a more computationally efficient analogue of the IBM: a stochastic compartment model (SCM) which followed daily cohorts of infected individuals through the same stages and fates. For extremely demanding computations, including Monte Carlo-based Bayesian inference, the SCM was approximated by a simpler deterministic compartment model (DCM) that follows daily cohorts through the same stages and fates, except that probabilistic transitions are replaced by proportional allocations.

The model suite represents the public health measures implemented in Australia in an attempt to prevent or limit outbreaks. These measures include: testing; contact tracing; self-quarantine/self-isolation of detected cases and contacts; border controls (self-quarantine and then hotel quarantine of overseas arrivals); and social distancing directives. Our model suite predicts the number of new infected cases each day as a result of transmission from current infectious cases, taking into account the effects of self-isolation and self-quarantine.

This section provides an overview of the model structure and function. Further details are provided in Appendix 1.

### Disease progression

**Fig. 1:**
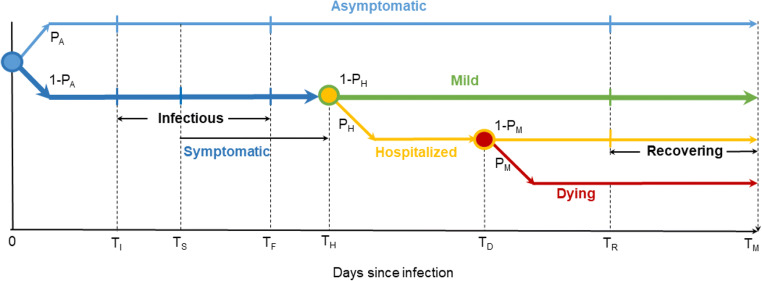
Model suite representation of COVID-19 progression

The model suite represents the key stages and possible outcomes in the evolution of COVID-19 infection, as portrayed in Fig. 1. Some asymptomatic COVID-19 cases never develop symptoms, but do become infectious, although they are believed to be less infectious than symptomatic cases. Newly infected cases in the model are immediately assigned to either an asymptomatic category (with probability P_A_) or a symptomatic category with probability (1-P_A_). Those in the symptomatic category do not develop symptoms until T_S_ days post-infection. The timing of the onset of symptoms matters because, at least during the period considered in this study, testing (and therefore detection, reporting and contact tracing) in Australia were mostly confined to those displaying symptoms.

Both asymptomatic and symptomatic categories become infectious after T_I_ days. Importantly, transmission can occur at least 1 to 2 days before symptoms became apparent (T_I_ < T_S_), hindering attempts to control COVID-19 outbreaks. In our model suite, the infectious period ends at T_F_ days, chosen so as to yield appropriate values for the reproductive number R0 and the growth rate of uncontrolled outbreaks.

A proportion of COVID-19 patients develop severe symptoms requiring hospital admission. In our model suite, this occurs after T_H_ days, with probability P_H_. Of those admitted to hospital, a further proportion will develop fatal complications, with probability P_M_. Those with fatal complications die with daily probability P_D_, starting T_D_ days after infection. All other patients start to recover with daily probability P_R_, beginning T_R_ days after infection. Our model suite only tracks cases for a maximum period of T_M_ days after infection after which cases have either recovered or died. Transitions in status related to onset of symptoms, onset and cessation of infectivity, and hospital admission occur simultaneously across all individuals in the same cohort. We developed versions of the models which allow these transitions to be spread over windows of multiple days noting that this made a negligible difference to our simulations.

### Control measures

Testing and detection of symptomatic cases occur in the model suite either through community testing, or through testing of those in self-quarantine or self-isolation. Community testing of cases self-reporting with symptoms was less effective in the first wave in Australia (March to May), as tests were in short supply and only contacts of overseas arrivals or known cases were tested. In Victoria’s second wave, attendance at community testing clinics was strongly encouraged, although some studies suggest only a modest proportion of those with COVID-19-like symptoms volunteered to be tested. In the model suite, the daily probability for a symptomatic case being tested and detected as positive at a community testing centre were allowed to increase from a low value of 0.2 in the first wave, to a higher value P_DC_ after June.

Testing and detection of symptomatic cases already in self-quarantine is assumed to be more effective, and their daily probability of detection P_DSQ_ is set to 0.8. The model assumes that all severe cases are tested and detected on admission to hospital.

Contact tracing has been a key and controversial public health measure against COVID-19 in Australia, and its representation in the model has been given particular attention. For each new infected case, the IBM keeps track of the ID of the responsible source case. If and when its source case is detected, an (undetected) case becomes eligible to be traced, with a daily probability P_T_. Once traced, cases are placed in self-quarantine noting that all detected cases are required to self-isolate.

The representation of contact tracing in the SCM and DCM is more challenging. In these models, only the numbers of cases in subcategories within a daily cohort are known. Our model suite tracks the proportions of detected cases within source cohorts, weighted by their relative contributions to each new cohort. They use this to calculate the proportion of members in each daily cohort which become subject to contact tracing. This calculation cannot replicate exactly the tracking of individual sources in the IBM, but provides a good approximation, allowing the SCM to closely replicate ensemble output from the IBM.

A highly effective contact tracing program would be expected to have values of the 24 h tracing efficiency P_T_ close to 1. Simulations show that contact tracing can still be highly effective with much lower values of P_T_ because the proportion of detected cases builds cumulatively over time. In the Victorian second wave, contact tracing by itself was insufficient to stop growth in infections and a severe lockdown was eventually imposed. We reproduced this fact in the model suite by assuming that a proportion P_U_ of infected cases were permanently undetected and untraceable.

Contact tracing is resource intensive, especially as the number of cases increases. For each detected case, a number of downstream contacts of order 10 must be identified, contacted, asked to self-quarantine, and monitored for development of symptoms, and to check compliance, for 14 days. Thus, for 100 daily detected cases, contact tracers could have up to 14,000 contacts under management. The Australia-wide tracing capacity T_CAP_ is assumed to be between 100 and 500 daily detected cases but we note that in specific jurisdictions, such as Victoria during its second wave, the capacity could have been below 100 new daily cases, in the absence of end-to-end automated process for enabling and recording contact tracing (Legal and Social Issues Committee 2020).

Australian border controls are implemented in the model as quarantine requirements on overseas arrivals. After 17 March 2020, overseas arrivals were required to self-quarantine at home. After 28 March they were required to enter hotel quarantine. The model simulations provided here are driven by reported numbers of daily detected COVID-19 cases among overseas arrivals in Australia. Positive overseas arrivals in quarantine are assumed to be detected immediately upon displaying symptoms and are represented in the model as new infectives appearing T_S_ days before being reported. Reported overseas cases are assumed to be accompanied by additional undetected asymptomatic cases, in the ratio P_A_:1–P_A_.

Social distancing (SD) or lockdown measures have played a key role in controlling both the first and second waves in Australia. The model suite represents SD implicitly as changes in the effective daily transmission rate G (new infections per infectious case per day). The maximum transmission rate in the absence of social distancing is denoted by G0. The minimum transmission rate achieved during the severe nation-wide lockdown in April 2020, which ended the first wave, is denoted by G_LD_. Movement data suggest there was a gradual relaxation following the first wave, up until early July. The extent of this relaxation of SD in early July is measured by the parameter RSD given by (G–GLD)/(G0–GLD). In the control scenarios described later, social distancing stringency is characterised by the control variable SD = (G0–G)/(G0–GLD).

### Transmission

In the IBM, the number of daily new infections produced by an infected individual is assumed to be a random variable drawn from an over-dispersed negative binomial distribution with mean G and dispersion coefficient k’. In the SCM, the number of daily new infections from a pool of X infectious individuals is then also negative binomial, with adjusted parameters G. X and k’.X. The choice of the dispersion parameter k’ is discussed further in the Appendix. In the DCM, the number of new daily infections is just the expected value G.X.

Individuals have different transmission rates, depending on their status. Cases in the asymptomatic category are assumed to have a mean transmission rate equal to F_A_ times that of those in the symptomatic category. While there should be zero transmission from cases in self-isolation or self-quarantine, in practice some transmission occurs. The model suite assumes the transmission rate from those in self-quarantine or self-isolation is reduced by the ‘leakage’ factor P_L_.

Hotel quarantine was initially assumed to be 100% effective in preventing transmission. However, cases of transmission from within hotel quarantine contributed to Victoria’s second wave, and multiple other cases of transmission from within hotel quarantine have been observed since in Australia. Accordingly, the model allows the generation of community infected cases from within hotel quarantine with a (very low) daily probability P_Q_.

All the models used in this study assume homogeneous mixing of infected with a susceptible pool of size SUS. The number of susceptible people, POP, is initially set to 20 million, assuming approximately 80% of the Australian population was initially susceptible. New infected cases are subtracted daily from SUS, and the daily transmission rate is multiplied by the fraction (1–SUS/POP). Given the small size of Australian outbreaks to date, the reduction in the susceptible pool size has negligible effect on transmission rates in the simulations presented here.

### Parameter uncertainty and Bayesian inference

The model suite has 24 model parameters (Table 1). In July–August 2020, when this study was undertaken, many of these parameters, particularly those related to the natural history of the disease, were considered to be well-constrained by prior knowledge. But others, particularly those defining the effectiveness of Australian control measures, were poorly constrained. We wanted to understand the capacity of the model to reproduce Australian observations of daily cases prior to that time, and the extent to which those observations could constrain the uncertain parameters, so as to reduce uncertainty in model simulations of responses to future control measures.

**Table 1:** Model parameters, prior values, and prior ranges and maximum likelihood values for those parameters subject to Bayesian inference

Symbol	Description	Prior value(s)	Maximum likelihood value
T_S_	Days to onset of symptoms	5 days	
T_I_	Days to onset of infectivity	4 days	
T_F_	Days to cessation of infectivity	8 days	
T_H_	Days to develop severe symptoms	10 days	
T_D_	Days to first deaths	12 days	
T_R_	Days to first recovery	19 days	
T_M_	Maximum period cases are active	40 days	
P_A_	Probability cases are asymptomatic	[0.1 0.4]	0.32
P_H_	Probability of hospitalisation for symptomatic cases	0.1	
P_M_	Probability of death among hospitalised cases	0.11	
P_D_	Daily probability fatally ill die after TD	0.15	
P_R_	Daily probability of recovery after TR	0.2	
G0	Daily transmission rate before social distancing	[0.3 0.65]	0.5
G_LD_	Daily transmission rate at peak of March–April lockdown	[0.05 0.25]	0.11
RSD	Relaxation of social distancing = (G–G_LD_)/(G0–G_LD_)	[0.7 1.0]	0.98
F_A_	Ratio of asymptomatic to symptomatic transmission	[0.1 0.4]	0.19
P_DC_	Daily probability of detection in community	[0.2 0.5]	0.36
P_DSQ_	Daily probability of detection in self-isolation	0.8	
P_T_	Daily probability of tracing downstream contacts	[0.2 0.8]	0.24
P_L_	Daily probability of transmission from self-isolated cases	[0.1 0.3]	0.11
P_U_	Fraction of community hidden/uncooperative	[0.1 0.6]	0.39
P_Q_	Daily probability of quarantine breakdown	0.0 to 0.01	0
POP	Total population size	20,000,000	
TCAP	Maximum tracing capacity in daily new cases	100–500	500

A Monte Carlo Bayesian inference procedure was, therefore, undertaken to fit the model to Australian observations obtained from https://www.covid19data.com.au/ and https://www.worldometers.info/coronavirus/#countries for the period 20 February to 5 July 2020, noting that estimation was completed 6 August.

A simple sample importance resample (SIR) procedure was used to obtain the posterior distribution of parameters. An ensemble of 200,000 simulations was generated, using independent random samples from the prior parameter distribution (the prior was treated as uniform on the parameter ranges specified in Table 1, and parameters were treated as independent in the prior). Because of the large ensemble size, the DCM was used as a fast approximation to the SCM in this procedure. Comparison of posterior ensembles from the SCM and DCM suggested this provided a good approximation to the posterior parameter distribution for both models. Both SCM and DCM were able to reproduce the observed time series well. (See Appendix 2 for detailed methods and results.)

Table 1 gives the maximum likelihood values of the uncertain parameters. The inference procedure yielded a large (10,000 member) random sample from the posterior parameter distribution. In the model scenarios described below, parameters for each simulation were drawn randomly from this posterior sample. This allowed us to represent the uncertainty in model predictions due to residual parameter uncertainty, as well as the uncertainty arising from stochastic events within the model itself.

### Model scenarios

Mandated social distancing measures were reintroduced on 9 July 2020 in Victoria, as rapidly growing daily case numbers reached a weekly average of 100. Model simulation scenarios (Table 2) were designed to assess the effectiveness of implementing different levels of social distancing at that trigger level. Social distancing levels in these scenarios are defined by the control variable SD = (G0–G)/(G0–GLD), so SD = 0 corresponds to no social distancing, and SD = 1 corresponds to the lockdown obtaining in April 2020.

**Table 2:** Median (2.5%–97.5 CI) values of additional Elimination days and Social distancing days (sum of social distancing level each day for 365 days) and number of COVID-19 deaths and associated (based on median values) Economy costs of social distancing, Value of statistical lives lost and Hospitalisation costs for social distancing levels SD from 0.5 to 1.0 for 365 days after implementation of social distancing when average daily cases over the preceding 7 days exceeds 100

Social distancing level	0.5	0.6	0.7	0.8	0.9	1.0
Elimination days (#)	360 (279–366)	366 (366–366)	249 (134–366)	118 (66–190)	73 (50–107)	51 (37–75)
Social distancing days (#)	183 (155–183)	220 (220–220)	196 (116–256)	117 (78–177)	94 (75–125)	83 (71–107)
Economy costs of social distancing (billion $)	38.43	46.2	41.16	24.57	19.74	17.43
COVID-19 deaths (#)	77,020 (53,822–104,277)	28,058 (448–69,931)	267 (135–1151)	135 (80–216)	101 (68–139)	86 (61–115)
Value of statistical lives lost ($ billion)	377.40	137.48	1.31	0.66	0.49	0.42

1. Economy costs of social distancing = $210 million per social distance day.

2. Value of statistical life = $4.9 million.

3. Elimination days is the number of days until zero community transmission (elimination) is achieved. Elimination days = 366 means the strategy fails to achieve no community transmission after 365 days

For each simulated SD level, from 0.5 to 1.0, mandated measures remain in place for a minimum 40-day period and then social distancing is relaxed in a linear fashion over 60 days. These scenarios assume highly effective border controls and quarantine for all new arrivals into Australia and P_Q_ is set to zero. Social distancing is not relaxed until there is no recorded community transmission. Thus, each of the six scenarios in Table 2 assumes the goal is to achieve no community transmission.

Two suppression scenarios were also simulated (see Table 3). In each suppression scenario, stringent social distancing measures (SD = 1.0) are imposed when the weekly average of new daily recorded cases is 100, but relaxation is triggered by a weekly average of 20 daily recorded cases. In suppression scenario A, social distancing is imposed for a minimum of 40 days before the relaxation criteria is assessed, while in suppression scenario B, there is no minimum period. For both scenarios, once the relaxation criteria are met, gradual relaxation to zero social distancing occurs over a 60-day period. In each scenario, border quarantine leakage (failure) occurs with a daily probability of 0.2% per infected arrival from overseas (P_Q_ = 0.002).

**Table 3:** Median (2.5%–97.5 CI) values of additional Social distancing days (sum of social distancing level each day for 365 days) and number of COVID-19 deaths and associated (based on median values) Economy costs of social distancing, Value of statistical lives lost and Hospitalisation costs for social distancing level = 1.0 for 365 days after implementation of social distancing

	Suppression scenario A^3^	Suppression scenario B^4^
Social distancing days (#)	101 (71–210)	115 (52–225)
Economy costs of social distancing (billion $)	21.21	24.15
COVID-19 deaths (#)	124 (66–261)	190 (67–411)
Value of statistical lives lost ($ billion)	0.61	0.93

1. Economy costs of social distancing = $210 million per social distance day.

2. Value of statistical life = $4.9 million.

3. Social distancing is implemented for 40 days after which gradual relaxation over 60 days occurs once the weekly average of new daily recorded cases declines to 20.

4. No minimum of 40 days of social distancing; gradual relaxation over 60 days occurs once the weekly average of new daily recorded cases declines to 20

For each of these scenarios, the SCM was used to generate an ensemble of 1000 runs, drawing parameter sets randomly from the posterior distribution produced by the Bayesian inference procedure described above. The simulated ensemble outputs were statistically analysed and daily percentiles calculated.

### Economy costs

Economy-wide costs of the national and high stringency social distancing that began in March 2020 are based on Australian Bureau of Statistics (ABS) data at a Victorian level equivalent to approximately $210 million per lockdown day (Kompas et al. 2020). Economy costs of a lockdown were assumed to be linear in the different levels of mandated social distancing, noting that greater social distancing and an increased frequency of cycles of high stringency social distancing followed by relaxation are likely to more than proportionally increase economy costs. COVID-19 related fatalities are valued at $4.9 million per value of statistical life (VSL), sourced from Prime Minister and Cabinet (Prime Minister and Cabinet 2020).

## Results and discussion

### Effects of social distancing stringency in elimination scenarios

**Fig. 2:**
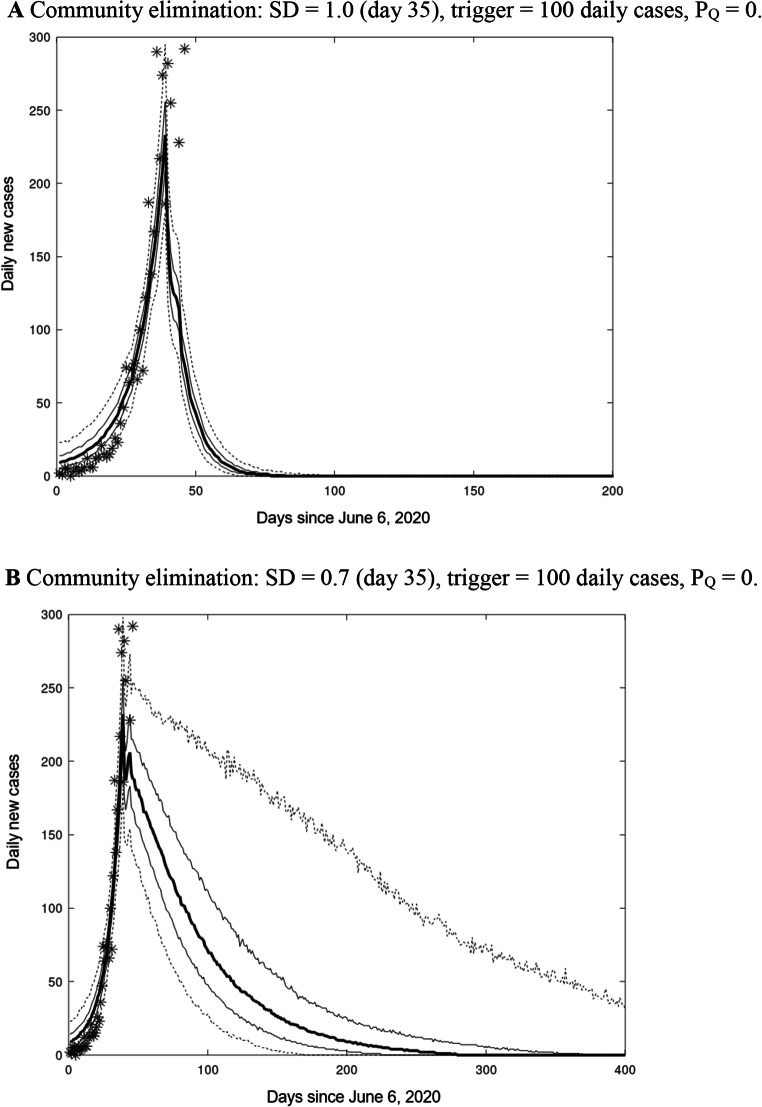
**a** Community elimination: SD = 1.0 (day 35), trigger = 100 daily cases, P_Q_ = 0. **b** Community elimination: SD = 0.7 (day 35), trigger = 100 daily cases, P_Q_ = 0. N.B: Simulations (median, quartiles, 5–95 percentiles) are from a 1000 members ensemble and observed daily new local Australian cases for SD levels. Median (thick line), quartiles (thin lines), 5–95 percentiles (dashed lines), observed daily new Australian local cases, 6 June to 15 July 2020 (*)

Results are provided in Table 2 for a period of 365 days following initial implementation of different levels of SD, assuming no leakage from hotel quarantine. Stringent social distancing, (SD = 1.0), results in elimination of community transmission after approximately 50 days (median), and within 80 days for every simulation (Fig. 2). By comparison, moderate social distancing (SD = 0.7) takes some 250 days (median) to achieve community elimination and 21% of simulations fail to eliminate community transmission within one year. Stringent social distancing (SD = 1) results in economy costs of $17.4B compared to $41.2B with SD = 0.7. (Table 1, Fig. 2). The scenarios with weaker social distancing (SD of 0.5 and 0.6) result in uncontrolled COVID-19 outbreaks. For SD of 0.5, some ensemble members achieve elimination within 365 days through herd immunity but at the loss of between 54,000 and 104,000 lives (Table 1).

### Effect of relaxation rules in suppression scenarios

**Fig. 3:**
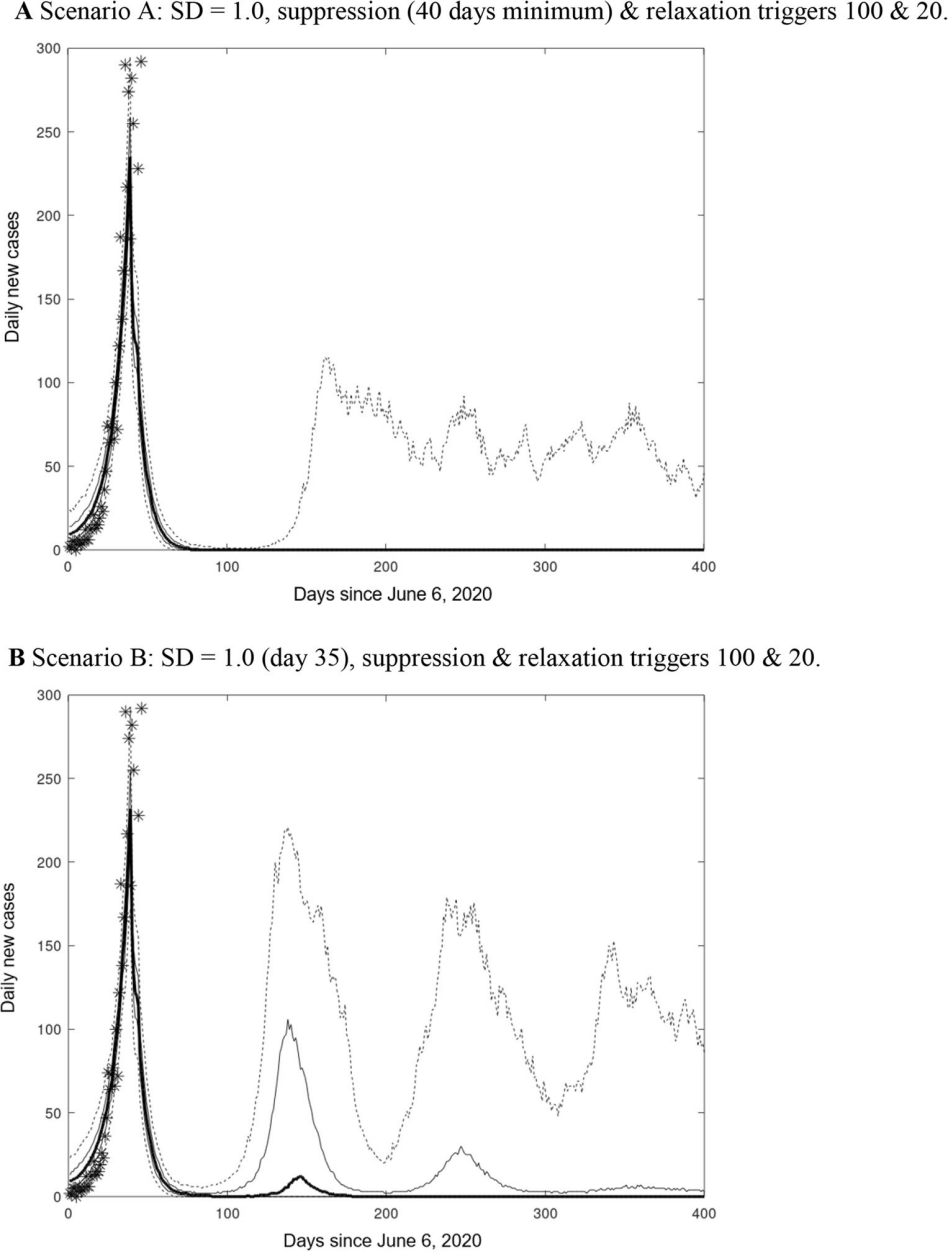
**a** Scenario A: SD = 1.0, suppression (40 days minimum) & relaxation triggers 100 & 20. **b** Scenario B: SD = 1.0 (day 35), suppression & relaxation triggers 100 & 20. N.B. Ensemble percentiles: median (thick line), quartiles (thin lines), 5–95 percentiles (dashed lines), observed daily new Australian local cases, 6 June to 15 July 2020 (*). Triggers defined by daily cases. SD begins at day 35. Quarantine leakage, P_Q_ = 0.002 in 2a and 2b

Figure 3a,b compares the simulated daily new cases (median, quartiles, 5–95 percentiles from a 1000 member ensemble) with observations for suppression scenario A (with a minimum 40 days implementation of social distancing) and suppression scenario B without a minimum duration constraint. For suppression scenario A, elimination of community transmission is achieved in more than 75% of ensemble members, and the ensemble median achieves and maintains elimination within 100 days (Table 3). For suppression scenario B, there is a greater than 50% chance of a second outbreak requiring reimposition of social distancing, and a greater than 25% chance of further cycles of outbreak and social distancing (Fig. 3b). The median time to community elimination is twice that for suppression scenario A (Table 3).

Even for suppression scenario A, more than 5% of the ensemble members display continuing outbreaks. The seeding of new infectives by rare and random failures of hotel quarantine (P_Q_ = 0.002) may contribute, but the quasi-cyclic nature of these outbreaks suggests there is a small but non-zero risk that relaxation after 40 days, at a trigger level of 20 daily cases, fails to achieve community elimination. This risk is clearly much greater for suppression scenario B, which allows relaxation of social distancing within 40 days.

### Limitations of this study

Our model suite does not allow us to fully capture the differences in transmission across multiple communities or sub-populations as would an agent-based model. Such transmission differences may arise from multiple factors, including cultural reasons, housing density, and the proportion of workers who are in the casual workforce and who may have financial incentives not to be tested or go to work sick.

The relationship between hidden transmission and essential workers, not accounted for in our modelling, is relevant for the effectiveness of social distancing. This is because, depending on the stringency of social distancing measures, workers may still be able to infect their workmates at their workplaces. In recognition of this incentive problem, supplementary payments of A$1500 were provided to Victorian workers from 20 June 2020 who tested positive or who were a close contact to someone who tested positive, and from 13 August, A$450 was provided to those who were in self-isolation awaiting test results.

## Conclusions

Our results provide robust support for a highly stringent suppression strategy in relation to COVID-19 infections in Australia. We find: one, that better public health outcomes (reduced COVID-19 fatalities) are positively associated with lower economy costs and higher levels of social distancing; two, achieving zero community transmission lowers both public health and economy costs compared to allowing community transmission to continue; three, early relaxation of social distancing, and in particular in the absence of a minimum social distancing period (minimum 40 days) and with quarantine leakage, increases both public health and economy costs; four, our simulated local cases using data until 17 July, are comparable to the actual suppression of COVID-19 in Victoria after 5 August that had the implicit goal of community elimination and gradually relaxed SD measures at pre-defined thresholds.

If the goal of social distancing is to achieve zero community transmission (elimination), as in the State of Victoria, SD levels of 0.8, 0.9 and 1.0, achieve elimination with a 100% probability over the 365 days. SD levels of 0.5 and 0.6 fail to achieve community elimination within the simulation period. A SD level of 0.7 achieves elimination within 365 days in approximately 80% of simulations. Lower levels of SD *increase* both COVID-19 fatalities and economy costs. This finding is consistent with an agent-based model for Victoria that compares a standard lockdown (with and without masks) with a more severe lockdown (Blakely et al. 2020) and a national model developed for the first wave of COVID-19 infections in Australia (Chang et al. 2020).

If suppression (rather than elimination of community transmission) is the goal, such that relaxation of social distancing measures begins at a threshold relating to the weekly average of new daily recorded cases, then lower costs are incurred when social distancing is imposed for a minimum period that is sufficiently long. Imposing a binding minimum number of social distancing days per lockdown reduces the total days in lockdown over a 12-month period and, thus, the associated economy costs.

## Figures and Tables

**Fig. 4:**
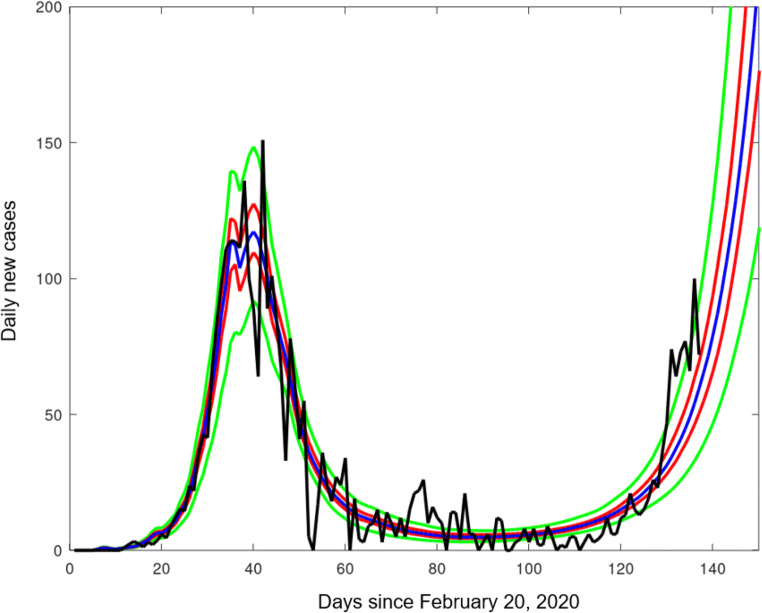
Daily new Australian cases of local origin from 20 February 2020. Observations in black. Percentiles for a posterior ensemble from the DCM: median (blue), quartiles (red), 2.5 and 97.5 percentiles (green)

**Fig. 5:**
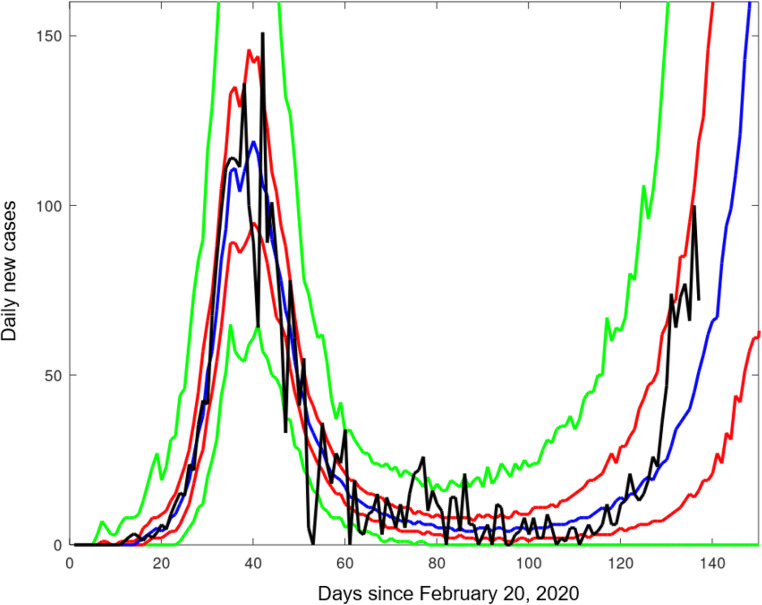
Daily new Australian cases of local origin from 20 February 2020. Observations in black; percentiles for a posterior ensemble from the SCM: median (blue), quartiles (red), 2.5 and 97.5 percentiles (green)

**Fig. 6:**
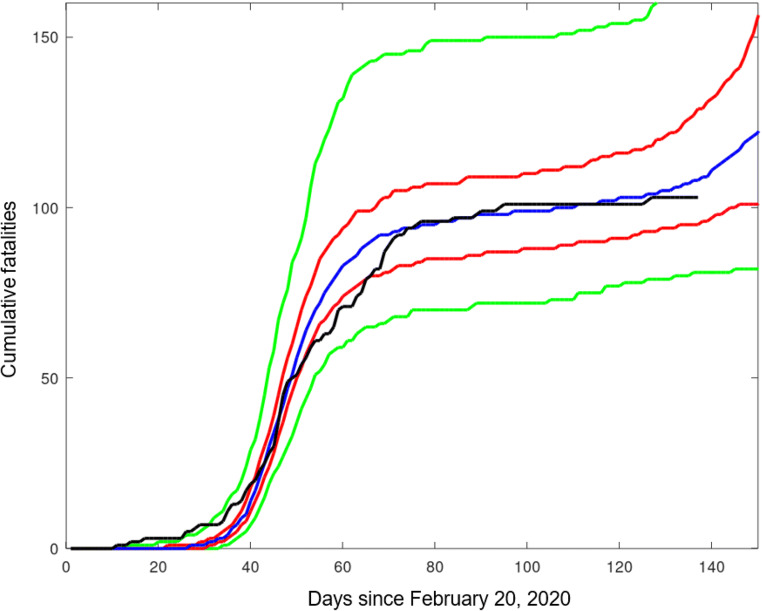
Cumulative COVID-19 fatalities for Australia starting 20 February 2020. Observations in black; percentiles for a posterior ensemble from the SCM: median (blue), quartiles (red), 2.5 and 97.5 percentiles (green)

**Fig. 7:**
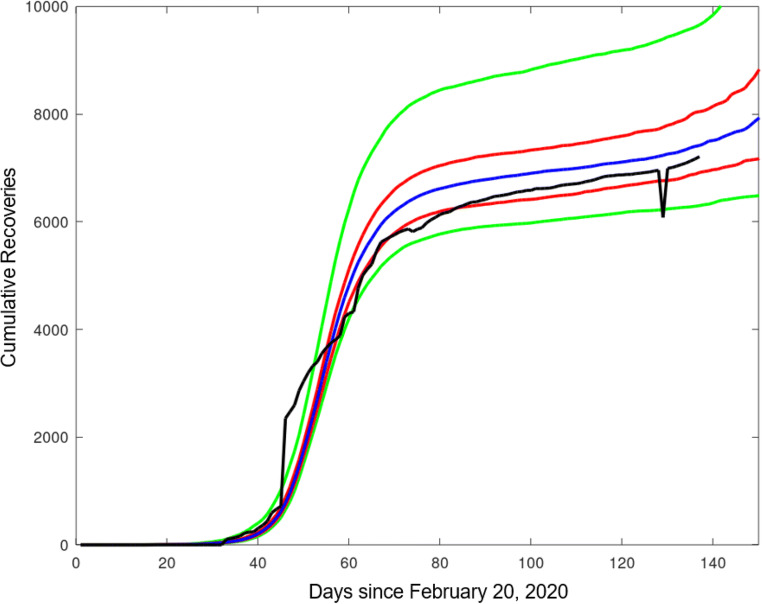
Cumulative COVID-19 recoveries for Australia starting 20 February 2020. Observations in black; percentiles for a posterior ensemble from the SCM: median (blue), quartiles (red), 2.5 and 97.5 percentiles (green)

**Fig. 8:**
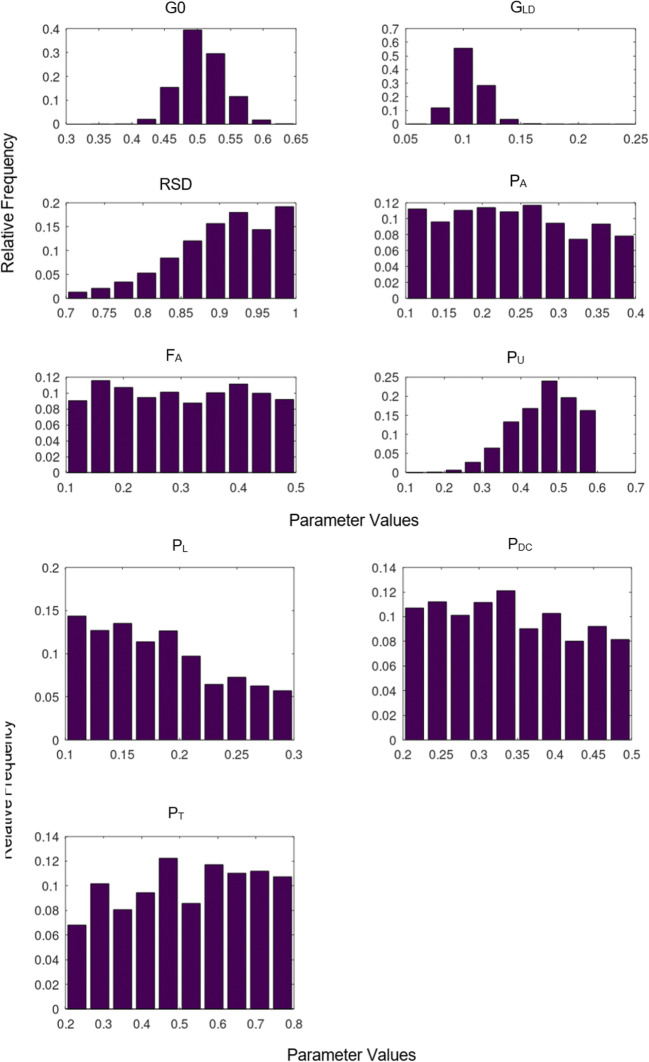
**a** Marginal posterior pdfs for parameters subject to Bayesian inference. **b** Marginal posterior pdfs for parameters subject to Bayesian inference

**Fig. 9:**
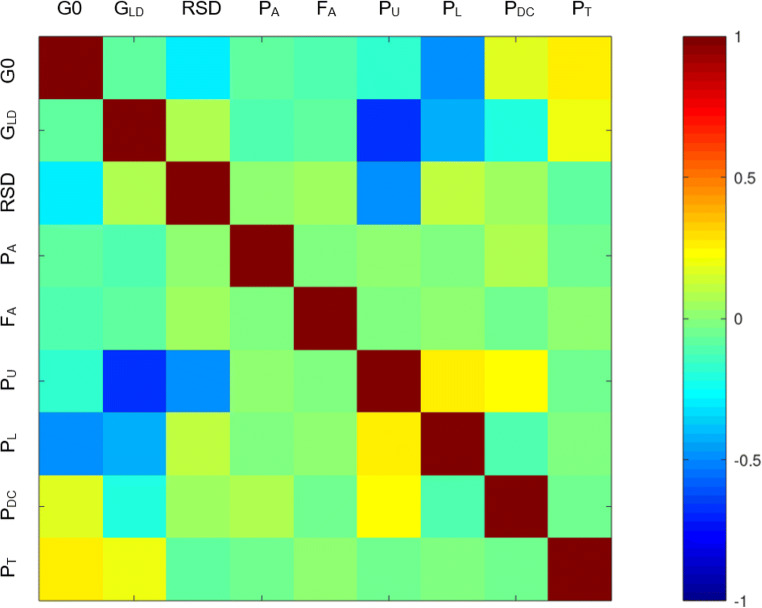
Posterior correlation structure among parameters subject to Bayesian inference

## Data Availability

Can be accessed at https://osf.io/2r9h6/

## Appendix 1

### Detailed model formulation

The individual-based model (IBM) represents individual infected cases, which are assigned a unique sequential ID. Changes in the status of each case are recorded as changes in an array of quality attributes attached to each ID. The stochastic compartment model (SCM) represents numbers of individual cases in daily cohorts of newly infected people. It indexes daily cohorts by the day d they were infected. The SCM must represent changes in status by dividing each daily cohort into a set of labelled sub-compartments, with one sub-compartment for each meaningful and feasible combination of attributes. Changes in the status of individuals with respect to disease progression, detection, contact tracing and quarantine are recorded by transferring appropriate number of individuals between sub-compartments.

The sub-compartments and associated labels for the SCM are given in Table 4. These are sub-compartments of daily cohorts; for example, for label SF, X_SF_(t,d) is the number of cases in sub-compartment SF on day t within the cohort infected on day d. The two exceptions are the compartments for the dying and recovering: these are pooled across daily cohorts; therefore, X_FI_(t) and X_RD_(t) represent the total number of individuals across all relevant cohorts who are respectively dying or recovering on day t.

**Table 4:** Sub-compartments within daily cohorts in the SCM

Label	Description
SF	Symptomatic/pre-symptomatic in community
AF	Asymptomatic in community
SU	Hidden symptomatic/pre-symptomatic
AU	Hidden asymptomatic
ST	Traceable symptomatic/pre-symptomatic in community
AT	Traceable asymptomatic in community
SSQ	Symptomatic/pre-symptomatic in self-quarantine
ASQ	Asymptomatic in self-quarantine
D	Detected (symptomatic) cases
SFO	Overseas pre-symptomatic in community
SSQO	Overseas pre-symptomatic in self-quarantine
SQ	Overseas symptomatic/pre-symptomatic in hotel quarantine
AQ	Overseas asymptomatic in hotel quarantine
SH	Hospital patients
FI	Fatally ill hospital patients
M	Dead
RD	Recovering detected cases

The DCM has the same sub-compartments as the SCM. The numbers of individuals in sub-compartments in the SCM are integers, and transfers between sub-compartments are determined by drawing integer numbers from appropriate probability distributions. In contrast, the numbers in sub-compartments in the DCM are rational numbers, and transfers are computed based on expected fractions.

The transitions in status associated with disease progression, detection, contact tracing and quarantine are described qualitatively in the body of the paper. For the most part, the implementation of these as transfers between sub-compartments in the SCM is straightforward. Whenever a set of X individuals each face a probability P of change in status the model transfers DX individuals to the corresponding sub-compartment, with DX drawn from a binomial distribution with parameters X and P. If there are more than two possible outcomes, a multinomial distribution is used. The equations and conditions for probabilistic transfers among sub-compartments are spelt out in detail in Table 5, with the exceptions of transmission and source detection. These are implemented as follows.

**Table 5:** Sub-compartment exchanges in SCM, in order of execution in each daily time step

Process	Daily cohorts	Compartment exchanges
Transmission	T_I_ < = d < = T_F_	See Text.
Asymptomatic & hidden	d = 0	Given X new local cases: [X_SF_, X_AF_, X_SU_, X_AU_] is ~ multinomial (X, [(1-P_A_).(1-P_U_), P_A_.(1-P_U_), P_U_(1-P_A_), P_A_.P_U_])
Overseas arrivals	d = 0	Overseas reported (symptomatic) cases XSO treated as newly infected T_S_ days earlier. Matched by asymptomatic cases XAO ~ BIN(XSO,P_A_/(1-P_A_)). For arrivals: Before March 17: X_SFO_ = XSO, X_AF_ = X_AF_ + XAO. From March 17 to March 28: X_SSQO_ = XSO, X_ASQ_ = XAO. After March 28: X_SQ_ = XSO, X_AQ_ = XAO.
Hotel quarantine breakdown	d = 0	Hotel quarantine evasions QE ~ BIN(XSQ,P_Q_). X_SQ_ = X_SQ_ – QE, X_SU_ = X_SU_ + QE.
Source detected	d > = 1	DXST = additional symptomatic cases with detected sources. DXAT = additional asymptomatic cases with detected sources. (See text for derivation). X_ST_ = X_ST_ + DXST, X_SF_ = X_SF_ – DXST. X_AT_ = X_AT_ + DXAT, X_AF_ = X_AF_ – DXAT.
Contact tracing	d > = 1	CST ~ BIN(X_ST_,P_T_). X_ST_ = X_ST_ – CST, X_SSQ_ = X_SSQ_ + CST. CAT ~ BIN(X_AT_,P_T_). X_AT_ = X_AT_ – CAT, X_ASQ_ = X_ASQ_ + CAT.
Detection of overseas cases	d = T_S_	X_D_ = X_D_ + X_SFO_ + X_SSQO_, X_SFO_ = 0, X_SSQO_ = 0. YDET = YDET + X_SFO_ + X_SSQO_ + X_SQO_
Detection of cases in self-quarantine	d > = T_S_	DSQ ~ BIN(X_SSQ_,P_DSQ_). X_SSQ_ = X_SSQ_ – DSQ, X_D_ = X_D_ + DSQ. YDET = YDET + DSQ, YDETL = YDETL + DSQ.
Detection of cases in community	d > = Ts	DSF ~ BIN(X_SF_,P_DC_). X_SF_ = X_SF_– DSF, X_D_ = X_D_ + DSF. DST ~ BIN(X_ST_,P_DC_). X_ST_ = X_ST_– DST, X_D_ = X_D_ + DST. YDET = YDET + DSF + DST, YDETL = YDETL + DSF + DST.
Hospitalisation	d = T_H_	For each of X = X_SF_, X_SSQ_, X_ST_, X_SU_, X_D_, X_SQ_ DH ~ BIN(X,P_H_). X_SH_ = X_SH_ + DH, YH = YH + DH. For CC = SF, SSQ, ST, SU, D: X_CC_ = X_CC_ – DH. For CC = SF, SSQ, ST, SU: YDET = YDET + DH, YDETL = YDETL + DH.
Fatally ill	d = T_D_	DFI ~ BIN(X_SH_,P_M_). X_SH_ = X_SH_ – DFI, X_FI_ = X_FI_ + DFI.
Death	T_D_ < = d < = T_M_	DD ~ BIN(X_FI_,P_D_). X_FI_ = X_FI_ – DD, X_M_ = X_M_ + DD. YM = YM + DD.
Recovery	T_C_ < = d < = T_M_	DRD ~ BIN(X_D_,P_R_). X_D_ = X_D_ – DRD, X_RD_ = X_RD_ + DRD. DRH ~ BIN(X_SH_,P_R_). X_SH_ = X_SH_ – DRH, X_RD_ = X_RD_ + DRH. YRD = YRD + DRD + DRH.

#### Transmission

Daily infections per individual are assumed to have a negative binomial distribution with mean G and dispersion parameter k’. Total infections per individual over an infectious period T_L_ (= T_F_ + 1-T_I_) will then be negative binomial with mean G.T_L_, and dispersion coefficient k = k’.T_L_. The sum of daily new infections from XI sources is then negative binomial with mean G.XI and dispersion coefficient k’.XI. It has been reported that total infections per individual are highly over-dispersed; therefore, k is set to 0.2. This means that most infected individuals infect no-one, while rare super-spreaders can infect 30 or more.

The number of potentially infectious sources on day t is obtained by summing cases from all potentially infectious cohorts X(t,d) with T_I_ < = t – d < = T_F_. These sources need to be divided into five classes, according to their contributions to transmission:

- Free symptomatic sources:
  $$ {\mathrm{S}}_{\mathrm{S}\mathrm{F}}\left(\mathrm{t}\right)={\sum}_{\mathrm{d}}\left\{{\mathrm{X}}_{\mathrm{S}\mathrm{F}}\left(\mathrm{t},\mathrm{d}\right)+{\mathrm{X}}_{\mathrm{S}\mathrm{U}}\left(\mathrm{t},\mathrm{d}\right)+{\mathrm{X}}_{\mathrm{S}\mathrm{T}}\left(\mathrm{t},\mathrm{d}\right)+{\mathrm{X}}_{\mathrm{S}\mathrm{O}}\left(\mathrm{t},\mathrm{d}\right)\right\},\mathrm{for}\ {\mathrm{T}}_{\mathrm{I}}<=\mathrm{t}\hbox{--} \mathrm{d}<={\mathrm{T}}_{\mathrm{F}}; $$
- Self-quarantined symptomatic sources:
  $$ {\mathrm{S}}_{\mathrm{S}\mathrm{SQ}}\left(\mathrm{t}\right)={\sum}_{\mathrm{d}}\ \left\{{\mathrm{X}}_{\mathrm{S}\mathrm{SQ}}\left(\mathrm{t},\mathrm{d}\right)+{\mathrm{X}}_{\mathrm{S}\mathrm{SQ}\mathrm{O}}\left(\mathrm{t},\mathrm{d}\right)\right\},\mathrm{for}\ {\mathrm{T}}_{\mathrm{I}}<=\mathrm{t}\hbox{--} \mathrm{d}<={\mathrm{T}}_{\mathrm{F}}; $$
- Free asymptomatic sources:
  $$ {\mathrm{S}}_{\mathrm{AF}}\left(\mathrm{t}\right)={\sum}_{\mathrm{d}}\ \left\{{\mathrm{X}}_{\mathrm{AF}}\left(\mathrm{t},\mathrm{d}\right)+{\mathrm{X}}_{\mathrm{AU}}\left(\mathrm{t},\mathrm{d}\right)+{\mathrm{X}}_{\mathrm{AT}}\left(\mathrm{t},\mathrm{d}\right)\right\},\mathrm{for}\ {\mathrm{T}}_{\mathrm{I}}<=\mathrm{t}\hbox{--} \mathrm{d}<={\mathrm{T}}_{\mathrm{F}}; $$
- Self-quarantined symptomatic sources:
  $$ {\mathrm{S}}_{\mathrm{ASQ}}\left(\mathrm{t}\right)={\sum}_{\mathrm{d}}\ {\mathrm{X}}_{\mathrm{ASQ}}\left(\mathrm{t},\mathrm{d}\right),\mathrm{for}\ {\mathrm{T}}_{\mathrm{I}}<=\mathrm{t}\hbox{--} \mathrm{d}<={\mathrm{T}}_{\mathrm{F}}. $$
- Self-isolating detected sources:
  $$ {\mathrm{S}}_{\mathrm{D}}\left(\mathrm{t}\right)={\sum}_{\mathrm{d}}\ {\mathrm{X}}_{\mathrm{D}}\left(\mathrm{t},\mathrm{d}\right),\mathrm{for}\ {\mathrm{T}}_{\mathrm{I}}<=\mathrm{t}\hbox{--} \mathrm{d}<={\mathrm{T}}_{\mathrm{F}}. $$

These sources are then weighted appropriately to calculate their expected contribution to transmission. Contributions from cases in self-isolation and self-quarantine at home are multiplied by the proportion P_L_ that breach self-isolation/self-quarantine. Contributions from asymptomatic community cases are multiplied by F_A_. These scaled contributions are then summed to produce an infectious potential on day t, XI(t):

$$ \mathrm{XI}\left(\mathrm{t}\right)={\mathrm{S}}_{\mathrm{S}\mathrm{F}}\left(\mathrm{t}\right)+{\mathrm{P}}_{\mathrm{L}}.{\mathrm{S}}_{\mathrm{S}\mathrm{SQ}}\left(\mathrm{t}\right)+{\mathrm{P}}_{\mathrm{L}}.{\mathrm{S}}_{\mathrm{D}}\left(\mathrm{t}\right)+{\mathrm{F}}_{\mathrm{A}}.{\mathrm{S}}_{\mathrm{A}\mathrm{F}}\left(\mathrm{t}\right)+{\mathrm{F}}_{\mathrm{A}}.{\mathrm{P}}_{\mathrm{L}}.{\mathrm{S}}_{\mathrm{A}\mathrm{SQ}}\left(\mathrm{t}\right). $$

#### Contact tracing

On day t, the cohort X(t,d) infected on day d has potential source cohorts X(t,d_1_) with T_I_ < = d– d_1_ < = T_F_. When the new cohort is formed on day d, the model computes and stores the total number of self-quarantining symptomatic sources S_SSQ_(d), and the total number of detected sources S_D_(d), summed over these potential source cohorts. On subsequent days t, the same calculation can be done over the updated source cohorts X(t,d_1_), to produce S_SSQ_(t,d), S_D_(t,d). Because detection is a random process in the SCM, we do not know exactly how many of the intervening detections, S_D_(t,d) – S_D_(d), come from self-quarantined sources vs free sources. However, given self-isolated symptomatic cases are detected with high probability, it is a reasonable approximation to assume the additional detections come first from the self-quarantined symptomatic source pool on day d, S_SSQ_(d), and after that from the free source pool. The decreases in self-quarantined sources and free sources from day d to day t are then given by:

$$ {\mathrm{D}\mathrm{EL}}_{\mathrm{S}\mathrm{Q}}\left(\mathrm{t},\mathrm{d}\right)=\min \left\{{\mathrm{S}}_{\mathrm{S}\mathrm{SQ}}\left(\mathrm{d}\right),{\mathrm{S}}_{\mathrm{D}}\left(\mathrm{t},\mathrm{d}\right)\hbox{--} {\mathrm{S}}_{\mathrm{D}}\left(\mathrm{d}\right)\right\}.{\mathrm{D}\mathrm{EL}}_{\mathrm{F}}\left(\mathrm{t},\mathrm{d}\right)={\mathrm{S}}_{\mathrm{D}}\left(\mathrm{t},\mathrm{d}\right)\hbox{--} {\mathrm{S}}_{\mathrm{D}}\left(\mathrm{d}\right)\hbox{--} {\mathrm{D}\mathrm{EL}}_{\mathrm{S}\mathrm{Q}}\left(\mathrm{t},\mathrm{d}\right). $$

The reduction in infectious potential since d is then:

$$ {\mathrm{DEL}}_{\mathrm{XI}}\left(\mathrm{t},\mathrm{d}\right)={\mathrm{DEL}}_{\mathrm{F}}\left({\mathrm{t}}_0,\mathrm{d}\right)+{\mathrm{P}}_{\mathrm{L}}.{\mathrm{DEL}}_{\mathrm{SQ}}\left(\mathrm{t},\mathrm{d}\right). $$

The fraction of cohort X(t,d) subject to downstream contact tracing on day t should then be:

$$ \mathrm{FT}\left(\mathrm{t},\mathrm{d}\right)={\mathrm{DEL}}_{\mathrm{XI}}\left(\mathrm{t},\mathrm{d}\right)/\mathrm{XI}\left(\mathrm{d}\right). $$

Individuals are moved incrementally from free to traceable compartments in each daily update. The fraction moved each day should be proportional to the daily increase in the traceable fraction, FT(t,d) – FT(t–1,d), with FT(d,d) = 0. The number to be moved is calculated as a proportion of the number remaining. Thus, the fraction moved each day from AF to AT and SF to ST is:

$$ \mathrm{FM}\kern0.5em =\left(\mathrm{FT}\left(\mathrm{t},\mathrm{d}\right)\hbox{--} \mathrm{FT}\left(\mathrm{t}-1,\mathrm{d}\right)\right)/\left(1\hbox{--} \mathrm{FT}\left(\mathrm{t}-1,\mathrm{d}\right)\right),\mathrm{provided}\ \mathrm{FT}\left(\mathrm{t}-1,\mathrm{d}\right)<1,=0\ \mathrm{if}\ \mathrm{FT}\left(\mathrm{t}-1,\mathrm{d}\right)=1. $$

The numbers moved each day are DXST = FM.XSF(t,d), DXAT = FM.XAF(d,t) (The SCM deals in integer numbers of cases;therefore, DXST and DXAT are rounded to the nearest integer. If either is less than 1, it is set to 1 with probability DXST or DXAT, zero otherwise).

## Appendix 2

### Bayesian Inference

#### Inference Methods

Australian observations were obtained from https://www.covid19data.com.au/ and https://www.worldometers.info/coronavirus/#countries for the period 20 February to 5 July 2020, noting that estimation was completed 6 August.

A simple sample importance resample (SIR) procedure was used to obtain the posterior distribution of parameters. The DCM was run using 200,000 independent random samples from the prior parameter distribution (the prior was treated as uniform on the parameter ranges specified in Table 1, and parameters were treated as independent in the prior). For each model run, a likelihood was calculated based on the SSQ errors between predictions and observations. To reduce any weekly reporting artefacts, and to allow for the DCM’s inability to reproduce any stochastic variation in the observations, the time series of observations and predictions were smoothed by a running 7-day average before calculating the SSQ error.

A ln(X + 10) transform was applied to the smoothed predictions and observations before computing the SSQ residuals, to give equal weight to errors at low and high case numbers, and to render the residuals approximately Gaussian. The likelihood was calculated from the SSQ assuming a Gaussian distribution of errors, with the degrees of freedom equal to the number of observations minus the number of parameters all divided by 7 to account for the effects of the 7-day running average. The error variance was estimated from the minimum SSQ corresponding to the maximum likelihood parameter set.

The resulting likelihoods were sorted into descending order, along with the associated parameter vectors, and effectively converted into a lookup table for a sample-based posterior cumulative probability density function. This allowed for straightforward random sampling from the posterior. Approximately 10,000 parameter vectors had non-negligible weight in the posterior.

#### Inference results

Percentiles of predicted daily detected local cases, based on an ensemble of 200 DCM trajectories using parameter sets drawn randomly from the posterior, are compared with the observations in Fig. 4. The fit is good, but we observe that the model does not adequately capture the steep rise in reported cases in the state of Victoria in late June – early July 2020. Percentiles for an equivalent ensemble from the SCM are shown in Fig. 5. The median predictions for the SCM and DCM agree closely. The SCM ensemble shows additional spread due to stochastic effects, and the observed steep increase in detected local cases in late June – early July 2020 does lie within the inter-quartile credibility interval for the stochastic model.

SCM posterior ensemble predictions of cumulative deaths are compared with observations in Fig. 6. The chosen parameter values for T_D_, P_M_ and P_D_ (Table 2) provide a good approximation to the timing and magnitude of observed cumulative deaths through the ‘first wave’. The second wave outbreak in Victoria that began in May 2020 started in a relatively younger demographic of workers and students, and only later spread into aged care homes beginning in July 2020 that resulted in over 90% of the Victoria second-wave fatalities.

The chosen values for parameters T_R_ and P_R_ (Table 2) leads to reasonable agreement in the timing of predicted and observed recoveries (Fig. 7), given some reporting anomalies in the observations. The median model prediction slightly over predicts recoveries, but given the good fit to daily cases, this may be because of incomplete official reporting of recoveries.

The marginal posterior pdfs for the parameters, calculated from the posterior weighted sample, are plotted as histograms in Fig. 8. The parameters fall into three groups with respect to the information provided by the observations. The parameters G0 and G_LD_ are highly informed, with posterior values restricted to a narrow range. The parameters RSD, P_U_ and P_L_ are moderately informed, with RSD biased to high values between 0.8 and 1, P_U_ to high values from 0.4 to 0.6 and P_L_ biased towards low values from 0.1 to 0.2. The remaining parameters seem to be effectively uninformed, with little difference between prior and posterior.

The posterior distribution carries additional information in the form of correlations among parameters (Fig. 9). A strong negative correlation between G_LD_ and P_U_ suggests the model is relying partly on contact tracing to bring about the steep decline in local cases in March–April 2020. High values of P_U_ (high levels of untraceable cases) weaken contact tracing, and more effective social distancing (lower G_LD_) is then required to match observations. There are also moderate negative correlations between RSD and P_U_, and between G0, G_LD_ and P_L_, with similar explanations. A weaker positive correlation between G0 and P_T_ suggests contact tracing reduces net transmissions prior to implementation of social distancing. The positive correlation between P_U_ and P_L_ may be an indirect result of their mutual strong negative correlations with transmission coefficients.

The DCM provides a relatively tight fit to observations despite some of the parameters being relatively poorly informed. This may be partly attributed to trade-offs among parameters, reflected in the correlation structure, although poorly informed and weakly correlated parameters such as P_A_ and F_A_ presumably have little influence on model predictions.

Our intent here was to provide decision-makers with a realistic picture of the uncertainty around the potential outcomes of alternative control strategies. We contend that scenario ensembles from the SCM, based on random samples from this posterior, provide a realistic picture of model uncertainty given prior knowledge and Australian observations to 5 July 2020.

## Code availability (software application or custom code)

Can be accessed at https://osf.io/2r9h6/
